# Differentiation of Benign Angiomatous and Microcystic Meningiomas with Extensive Peritumoral Edema from High Grade Meningiomas with Aid of Diffusion Weighted MRI

**DOI:** 10.1155/2014/650939

**Published:** 2014-11-16

**Authors:** Avetis Azizyan, Paula Eboli, Doniel Drazin, James Mirocha, Marcel M. Maya, Serguei Bannykh

**Affiliations:** ^1^Department of Radiology, Cedars-Sinai Medical Center, Los Angeles, CA 90048, USA; ^2^Department of Neurosurgery, Cedars-Sinai Medical Center, Los Angeles, CA 90048, USA; ^3^Biostatistics Research Institute, Cedars-Sinai Medical Center, Los Angeles, CA 90048, USA; ^4^Department of Pathology, Cedars-Sinai Medical Center, Los Angeles, CA 90048, USA

## Abstract

*Objective*. To determine whether angiomatous and microcystic meningiomas which mimic high grade meningiomas based on extent of peritumoral edema can be reliably differentiated as low grade tumors using normalized apparent diffusion coefficient (ADC) values. *Methods*. Preoperative magnetic resonance imaging (MRI) of seventy patients with meningiomas was reviewed. Morphologically, the tumors were divided into 3 groups. Group 1 contained 12 pure microcystic, 3 pure angiomatoid and 7 mixed angiomatoid and microcystic tumors. Group 2 included World Health Organization (WHO) grade II and WHO grade III tumors, of which 28 were atypical and 9 were anaplastic meningiomas. Group 3 included WHO grade I tumors of morphology different than angiomatoid and microcystic. Peritumoral edema, normalized ADC, and cerebral blood volume (CBV) were obtained for all meningiomas. *Results*. Edema index of tumors in group 1 and group 2 was significantly higher than in group 3. Normalized ADC value in group 1 was higher than in group 2, but not statistically significant between groups 1 and 3. CBV values showed no significant group differences. *Conclusion*. A combination of peritumoral edema index and normalized ADC value is a novel approach to preoperative differentiation between true aggressive meningiomas and mimickers such as angiomatous and microcystic meningiomas.

## 1. Introduction

Meningiomas are the most common intracranial tumor, comprising up to 20% of intracranial neoplasms [1]. The majority of meningiomas are benign with a classic appearance on CT and MRI. Less than 10% of meningiomas are atypical or anaplastic. The preoperative identification of these subtypes is important since they are more likely to recur and therefore require additional operative considerations [2–5]. Unfortunately, distinguishing benign meningiomas from atypical and anaplastic ones still remains unreliable on routine MRI [1–9]. For instance, the microcytic and angiomatous varieties of meningiomas are considered benign, WHO grade I tumors, but they resemble aggressive tumors on routine MRI [10]. On MRI, microcystic meningiomas are often hypointense on T1-weighted images and hyperintense on T2-weighted images and demonstrate various degrees of peritumoral edema. The latter feature raises a clinical suspicion for more aggressive tumors, including atypical/anaplastic meningiomas, hemangiopericytoma, solitary fibrous tumor, malignant gliomas, and metastatic carcinoma.

Apparent diffusion coefficient has emerged as a method for identifying high grade central nervous system tumors [11–13]. Several papers have specifically shown that high grade meningiomas also demonstrate low internal ADC [14–16]. To our knowledge ADC has not been utilized in conjunction with extent of peritumoral edema in order to identify low grade meningiomas which mimic high grade tumors.

Based on a review of the literature there are only four papers evaluating the MRI characteristics of microcystic and angiomatous meningiomas [10, 17–20]. In this study, we quantified the peritumoral edema as a ratio to tumor size. Additionally, we obtained global ADC values as well as tumor perfusion (CBV) data in order to determine if these three types of meningioma could be reliably differentiated preoperatively.

We find that CBV values are similar throughout all types of meningiomas and are therefore unreliable in differentiating low grade from high grade meningiomas. The peritumoral edema is elevated in microcystic and angiomatous meningiomas, with values similar to those seen in atypical and anaplastic meningiomas. However, the ADC values of microcystic and angiomatous meningiomas were similar to other types of WHO grade I meningiomas.

We propose that the utilization of these MRI characteristics is a novel approach that can assist neurosurgeons and radiologists in identifying low grade tumors which have aggressive MRI features.

## 2. Materials/Methods

### 2.1. Patient Selection

The Institutional Review Board at Cedars-Sinai Medical Center approved this study. Using PowerPath software we retrospectively screened surgical pathology database for meningiomas surgically treated at Cedars-Sinai Medical Center between November 2007 and March 2012. Subsequently we identified cases with predominantly microcystic and/or angiomatous morphology (group 1) and atypical and anaplastic meningiomas (group 2) and cases of benign meningiomas with fibrous and/or meningotheliomatous morphology (group 3). Histology of all tumors was reviewed by two or more neuropathologists independently.

In this study we mainly focused on MRI correlates of reactive changes in the brain parenchyma juxtaposed to a tumor. Because extensive peritumoral edema in groups 1 and 2 precluded a reliable analysis of the tumors adjacent to the spinal cord, sphenoid bone, cerebellum, and brainstem we therefore focused only on tumors of the cerebral convexities.

In our study, we selected 70 cases of meningioma. Group 1 included meningiomas of microcystic (*n* = 12), angiomatous (*n* = 3), and mixed angiomatous + microcystic (*n* = 7) morphology. Group 2 included atypical, WHO grade II (*n* = 28) and anaplastic, WHO grade III (*n* = 9) tumors, and group 3 was composed of WHO grade I meningiomas of meningotheliomatous (*n* = 8) and fibrous (*n* = 3) morphology [21].

**Figure 1:**
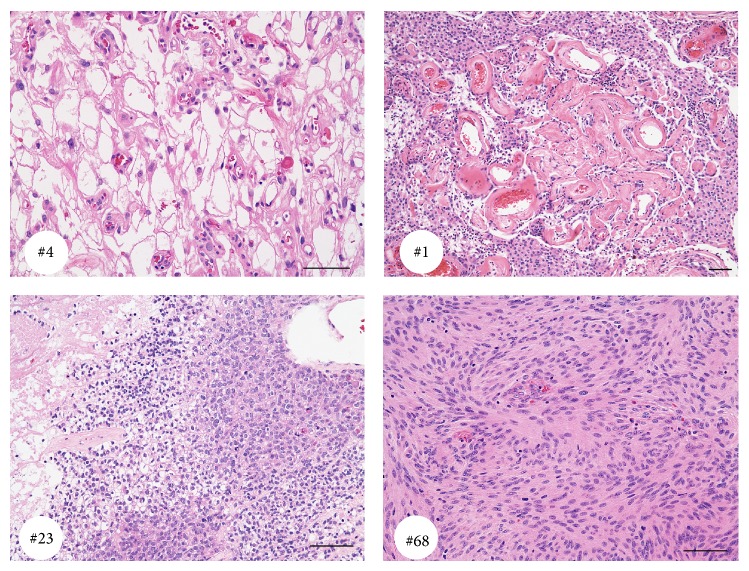
Representative images of three groups of meningiomas corresponding to case number as per the table in Supplementary Data (see Supplementary Material available online at http://dx.doi.org/10.1155/2014/650939). Microcystic meningioma (number 4), angiomatous meningioma (number 1), anaplastic meningioma (number 23), and fibrous meningioma (number 68). Bar is 100 *μ*m.

Twenty-five of our patients were male with age ranging from 30 to 91 years (the mean age was 60.6). The patients' demographics are presented in Table 1. Representative images of histopathology are shown in Figure 1. Characteristic MRI appearances of the different meningioma subtypes are presented in Figure 4.

### 2.2. MRI Imaging

Sagittal T1, transverse FLAIR, transverse T2, transverse DWI and ADC, transverse pre- and postcontrast T1 MRI, coronal postcontrast T1, CBV, and CBF were analyzed. Seventeen of the studies were performed on a 3.0 Tesla Siemens scanner, while the remaining cases were performed on 1.5 Tesla Siemens scanners (Erlangen, Germany). During the T1 contrast portion, 0.1 mmol per kilogram body weight of a gadolinium based contrast agent was administered intravenously. Postprocessing and normalization of the postcontrast images were performed to create cerebral blood volume (CBV) maps on separate Siemens workstations offline using Advance Neuro-Perfusion software (Erlangen, Germany). DWI was acquired and the ADC maps automatically generated by inline processing on the scanner console. The DWI sequence utilized a gradient echo planar pulse sequence (6000/109 [TR/TE]) and b values of 0 and 1000.

### 2.3. Data Analysis

#### 2.3.1. Peritumoral Brain Edema

**Figure 2:**
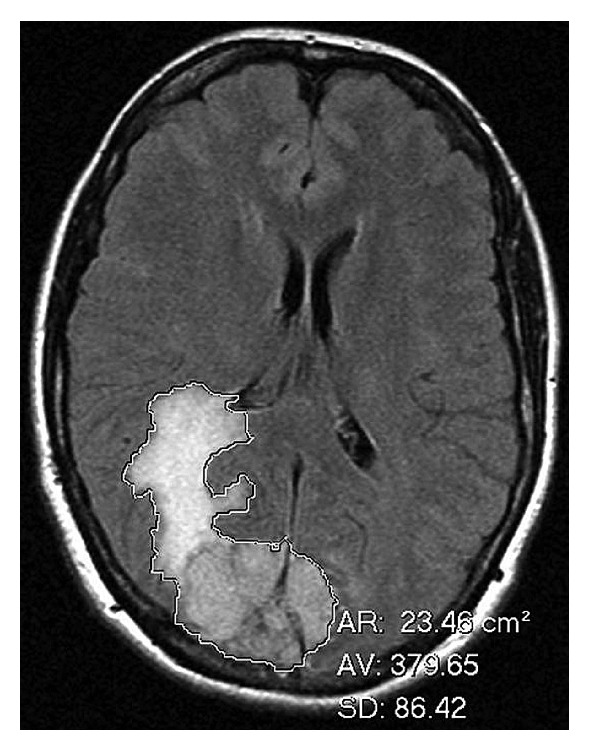
The tumor and adjacent edema is outlined with a freehand ROI tool. The surface area (AR) is provided in cm^2^.

The peritumoral brain edema index was defined as volume of tumor + volume of edema/volume of tumor. Volume measurements were obtained by using the freehand region of interest (ROI) tool on Kodak Carestream picture archiving and communication system (PACS) (Rochester, NY). After outlining the tumor and the tumor + edema on each slice of the FLAIR sequence a surface area was generated by the PACS (Figure 2). The surface area was multiplied by slice thickness to approximate the volume through that slice and all slice volumes were added to give total volume measurements.

#### 2.3.2. Perfusion and ADC

A global ADC of each meningioma was measured which served as an average ADC weighted based on tumor volume. An ROI was drawn around the tumor at every slice providing an average ADC at that slice. This was repeated at each slice and the PACS used to incorporate the data from multiple slices in order to produce a weighted average of the ADC. This standardized method prevents small regions, a particularly high or low ADC, from mischaracterizing the ADC of the remaining tumor. Global ADC of each meningioma was obtained by drawing ROI around the tumor at each slice. The ADC data was normalized by dividing these absolute ADC values by the ADC within the contralateral white matter. All studies had ADC sequences and 45 out of 70 had perfusion maps. Perfusion values were also obtained with a standard 30 mm^2^ ROI in a region of the highest intensity of CBV. The proportion of studies with perfusion data was equivalent among the different histological types.

### 2.4. Statistical Analysis

The peritumoral brain edema index, maximal perfusion, and maximal ADC values were compared between the different pathological types. The data set was assumed to be nonparametric as there was potential for nonnormal distributions. Therefore, significance was measured based on Kruskal-Wallis tests and set at *P* < 0.05.

## 3. Results

### 3.1. Peritumoral Edema

**Figure 3:**
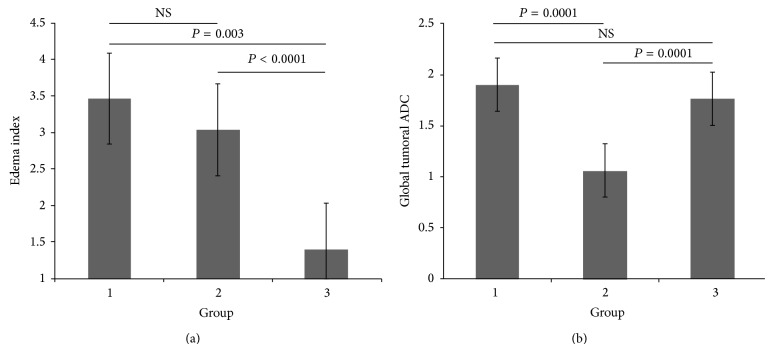
(a) Edema index mean compared between groups. There was no significant difference in the extent of peritumoral edema between groups 1 and 2 where group 1 consisted of angiomatous and microcystic tumors and group 2 consisted of WHO grades 2 and 3 meningiomas. The extent of peritumoral edema was significantly higher when comparing these higher grade (group 2) tumors to the WHO grade 1 tumors of group 3. Interestingly, there was a significantly larger extent of peritumoral edema in the WHO grade 1 meningiomas of group 1 when compared to 3. (b) Global tumoral ADC compared between groups. There was no significant difference in the global ADC values of the WHO grade 1 meningiomas in groups 1 and 3. Group 2 which had higher grade meningiomas had significantly lower ADC values when compared to either low grade group.

**Figure 4:**
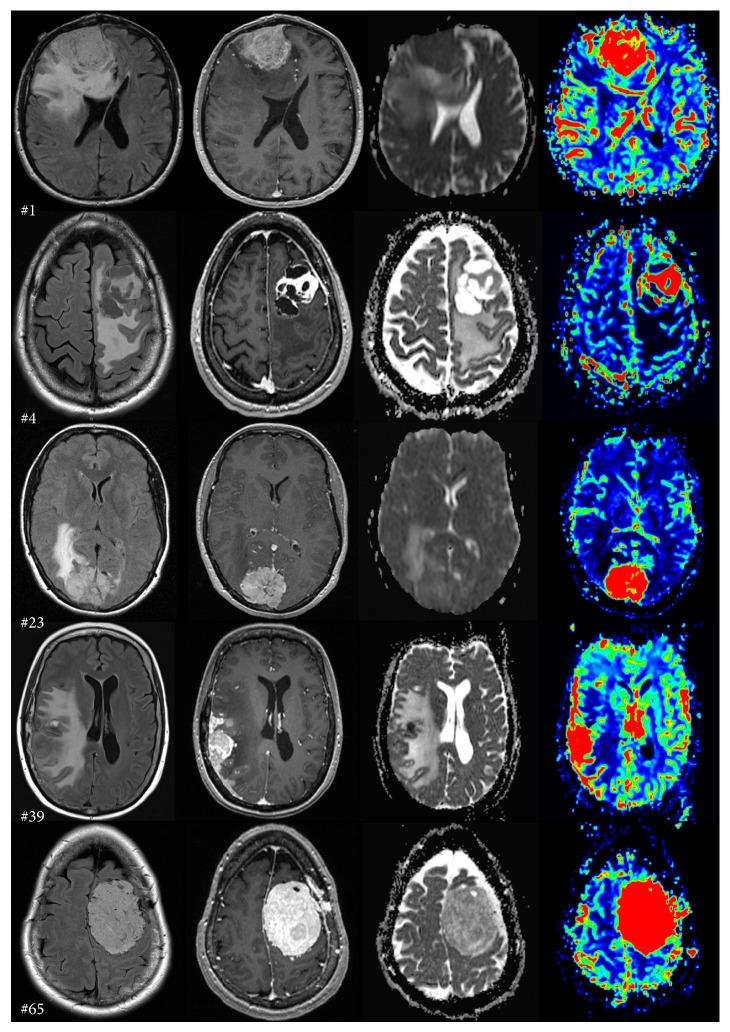
Characteristic MRI features. The first two rows are representative cases from group 1 (angiomatous, microcystic meningioma). The 3rd and 4th rows are group 2 meningiomas (anaplastic, atypical), whereas the last row is a meningotheliomatous tumor in group 3. The case number is shown in the bottom left corner of the FLAIR image. The columns are organized as FLAIR, T1WI + contrast, ADC map, and CBV map.

The edema index compares the volume of edema solicited by a meningioma based on the volume of the tumor. Both group 1 and group 2 meningiomas had significantly higher volume indices when compared to the low grade meningiomas in group 3 (*P* = 0.003 between groups 1 and 3, *P* < 0.0001 between groups 2 and 3) (Figure 3). When group 1 and group 2 were compared to one another there was no statistically significant difference, but mean edema index values were higher in group 1 (Table 1).

### 3.2. Perfusion

When comparing the highest cerebral blood volume based on ROI, there was no significant difference between the three groups. Mean values were higher in group 1, followed by groups 2 and 3, respectively (Table 1).

### 3.3. ADC

The global ADC values of the meningiomas as normalized to the contralateral white matter were compared. The values in the biologically high grade group 2 were significantly lower than either of the two low grade groups 1 and 3, with *P* values of 0.0001 and 0.0001, respectively. Groups 1 and 3 were not significantly different with respect to ADC value (Figure 3).

## 4. Discussion

Recent studies have identified similarities based on MRI appearance between WHO grade I angiomatous and microcystic meningiomas and more aggressive atypical and anaplastic meningiomas [10, 17–20]. In our study we analyzed peritumoral brain edema, ADC index, and perfusion values in order to differentiate low grade angiomatous and microcystic meningiomas from more biologically aggressive WHO grades II and III meningiomas and from other more common WHO grade I meningiomas of fibrous and meningotheliomatous morphology.

### 4.1. Peritumoral Brain Edema

Within the group of angiomatous and microcystic meningiomas peritumoral brain edema is reported with a prevalence ranging from 74 to 100% [10, 20, 22]. In our series, we found peritumoral brain edema in 100% of cases of angiomatous and mixed microcytic and angiomatous meningiomas. Within the histologically pure microcystic meningiomas we observed peritumoral edema in 66.6%. Interestingly, the rates of significant edema, as defined by a peritumoral edema index above 2, were similar between groups 1 and 2 (59%). In contrast, only 11% of meningiomas in group 3 had an edema index exceeding 2.

The WHO I, group 1 meningiomas of microcystic and angiomatoid histology showed similar prevalence and extent of edema as the anaplastic and atypical meningiomas. Furthermore, the edema index of these meningiomas was significantly higher than that found in other WHO grade I tumors in group III. Therefore, measurement of peritumoral brain edema cannot reliably separate biologically aggressive WHO grades II and III meningiomas from indolent meningiomas of angiomatous or microcystic histology.

Peritumoral brain edema has been observed in 40 to 60% of all meningiomas [22]. Kim et al. in their study of 86 meningiomas observed measurable peritumoral brain edema in 53.5% of WHO grade I and 80% of WHO grades II and III tumors [22]. Therefore, although high grade tumors are more likely to have peritumoral edema, more than half of WHO grade I tumors will also have accompanying edema.

Multiple contributing factors have been proposed including tumor size, histology, intratumoral venous congestion, and ischemia to adjacent brain due to tumor compression [23]. Of these, the overexpression and tumoral secretion of vascular endothelial growth factor (VEGF) has been shown to be most closely related to the extent of peritumoral edema [23–28]. VEGF is a potent inducer of capillary permeability and excessive cerebral and pial blood supply [24, 27, 28]. It is expressed by both low and high grade meningiomas leading to peritumoral edema [23]. It is not clear why low grade tumors also express VEGF. The fact that low grade tumors can express VEGF suggests that there is a more complex regulation of tumor angiogenesis than overexpression of VEGF alone. It has been shown that the ratio of VEGF to semaphoring 3A (SEMA3A), an antiangiogenic factor, correlates with tumor recurrence better than VEGF expression alone [29]. SEMA3A competes with VEGF for binding to tyrosine kinase receptor neuropilin-1 (NRP-1). Therefore, a low VEGF/SEMA3A ratio is seen in low grade tumors which typically do not have recurrence after total resection or progression to higher grade.

### 4.2. Apparent Diffusion Coefficient (ADC) Values

Multiple studies have correlated ADC values with tumor grade. Typically, tumors with higher cellularity and higher WHO grades show lower ADC values, as compared to normal brain tissue [11–13]. Studies looking specifically at meningiomas have found a similar correlation [14–16]. In their series of seventeen patients Filippi et al. found marked decrease in ADC values in atypical and anaplastic meningiomas, whereas benign meningiomas had values higher than normal brain parenchyma [14]. In our study we found that ADC intensities of angiomatous and microcystic meningiomas were similar to other WHO grade I tumors in group 3 but significantly higher (*P* < 0.0001) than those of group 2. Therefore, although microcystic and angiomatous tumors have aggressive appearing peritumoral edema, their internal ADC values are consistent low grade meningiomas.

### 4.3. Perfusion Values

Perfusion MRI is routinely used to assess the vascularity of meningiomas and in particular to differentiate between benign and malignant lesions [29, 30]. Zhang et al. observed no significant difference in perfusion values between malignant and benign meningiomas [29]. This is in concordance with our data, in which no difference in CBV was observed between the three groups. Despite slightly higher CBV values in group 1 as compared to group 3, the difference was not statistically significant.

## 5. Conclusion

Microcystic and angiomatous meningiomas present with aggressive appearing peritumoral edema similar to atypical and anaplastic meningiomas. This study demonstrates the potentially clinically valuable approach of utilizing ADC values to correctly and reliably identify them as distinguishing true high grade meningiomas from their mimickers.

## Supplementary Material

Summary table indicating patient age, sex and tumor histology. Degree of peritumoral edema, normalized ADC and perfusion values are provided for each patient. Patients are divided into Group 1, 2 or 3 based on histology. Group averages of the abovementioned data points are provided after each group.

## Figures and Tables

**Table 1:** Numerical summary for the three important outcome measures by group.

Group	*N*	Mean age (Std.)	MRI values	Number of cases with available data	Mean (Std.)	Upper 95% CI	Lower 95% CI
1	22	60.0 (14.9)	CBV perfusion	13	14.5 (6.1)	18.1	10.8
			Edema index	22	3.5 (3.1)	4.8	2.1
			ADC	22	1.9 (1.0)	2.3	1.5

2	37	60.7 (15.4)	CBV perfusion	22	12.4 (4.9)	14.6	10.2
			Edema index	37	3.0 (2.8)	4.0	2.1
			ADC	37	1.1 (0.2)	1.1	1.0

3	11	63.1 (15.0)	CBV perfusion	10	10.9 (3.1)	13.2	8.7
			Edema index	11	1.4 (0.7)	1.8	0.9
			ADC	11	1.8 (0.9)	2.4	1.2

Std. is standard deviation.
