# Supplementary material for: Differentiation of Benign Angiomatous and Microcystic Meningiomas with Extensive Peritumoral Edema from High Grade Meningiomas with Aid of Diffusion Weighted MRI
Source: Biomed Res Int. 2014 Nov 16;2014:650939. doi: 10.1155/2014/650939 (PMC4248374; doi:10.1155/2014/650939)
Supplement: Supplementary file 1 — Summary table indicating patient age, sex and tumor histology. Degree of peritumoral edema, normalized ADC and perfusion values are provided for each patient. Patients are divided into Group 1, 2 or 3 based on histology. Group averages of the abovementioned data points are provided after each group. [file 650939.f1.pdf]

Supplemental

Case#	Tumor type	Sex	Age	Location	Edema Index Vol edema/Vol tumor	Normalized tumor ADC	CBV Perfusion AV oval 30mm2
1	Angiomatoid	M	66	R frontal	4.4	1.2	11.3
2	Angiomatoid	F	68	L parietal	7.0	2.5	nd
3	Angiomatoid	M	50	L frontal	1.2	1.1	6.0
4	Microcystic	M	70	L frontal	4.4	3.4	26.0
5	Microcystic	F	63	Parafalcine	3.0	1.3	nd
6	Microcystic	F	60	L frontal	1.0	1.3	18.3
7	Microcystic	F	40	R frontal	1.1	3.9	15.0
8	Microcystic	M	70	R occipital	1.0	2.9	8.0
9	Microcystic	M	81	L frontal	4.2	1.5	25.2
10	Microcystic	F	78	Bifrontal	3.5	1.1	nd
11	Microcystic	F	67	R frotal	1.9	1.0	nd
12	Microcystic	F	88	R frontal	2.7	1.1	nd
13	Angiomatoid +microcystic	F	68	L frontal	1.0	1.4	15.0
14	Angiomatoid +microcystic	F	54	R temporal	1.7	1.8	11.3
15	Angiomatoid +microcystic	F	53	L frontal	2.5	1.4	15.0
16	Angiomatoid +microcystic	F	53	R frontal	6.4	1.7	7.8
17	Angiomatoid +microcystic	F	49	L frontal	5.7	4.5	16.0
18	Angiomatoid +microcystic	F	50	R frontal	1.2	1.7	nd
19	Angiomatoid +microcystic	F	43	R frontal	2.1	2.1	13.0
20	Angiomatoid +microcystic	M	36	R parietal	14.5	1.6	nd
21	Angiomatoid +microcystic	F	32	L frontal	3.8	1.9	nd
22	Angiomatoid +microcystic	F	74	L frontal	1.9	1.4	nd
	**Group Mean**		**60**		**3.5**	**1.9**	**14.5**
23	Anaplastic	F	50	R fronto-temporal	4.0	1.2	11.5
24	Anaplastic	M	65	L temp occipital	1.9	1.2	9.0
25	Anaplastic	F	50	R temporal	5.5	1.4	9.8
26	Anaplastic	M	65	L occipital	18.5	1.4	5.4
27	Anaplastic	M	86	R fronto-parietal	1.4	1.1	14.0
28	Anaplastic	F	50	R fronto-parietal	2.6	1.2	10.0
29	Anaplastic	F	49	R froto-parietal	3.6	1.3	12.5
30	Anaplastic	M	64	parietal occipital	2.7	1.1	nd
31	Anaplastic	F	85	R frontal	1.9	1.1	nd
32	Atypical	M	53	Olfactory groove	1.8	1.0	9.0
33	Atypical	M	61	R hemisphere	3.6	1.1	nd
34	Atypical	F	30	L frontal	3.4	1.1	7.0
35	Atypical	M	62	L temporal	2.7	1.2	12.0
36	Atypical	M	68	R frontal	3.9	1.0	14.7
37	Atypical	M	75	L frontal	1.9	1.1	18.7
38	Atypical	M	65	R hemisphere	3.8	0.9	7.0
39	Atypical	F	49	Occipital	2.6	1.3	17.9
40	Atypical	F	91	L frontal	1.3	1.0	8.6
41	Atypical	F	75	L temporal	3.8	1.2	20.0
42	Atypical	F	48	R frontal	1.9	1.1	19.0
43	Atypical	F	34	R frontal	1.6	1.1	nd
44	Atypical	F	69	R frontal	1.8	0.9	nd
45	Atypical	F	67	R temporal	1.7	0.6	19.0
46	Atypical	F	69	R frontal	2.0	1.0	20.0
47	Atypical	M	53	Frontal	1.4	0.9	4.3
48	Atypical	M	42	R frontal	1.7	1.2	nd

49	Atypical	F	87	L temporal	1.2	0.9	nd
50	Atypical	M	66	R frontal	3.3	1.1	11.0
51	Atypical	F	52	R temporal	1.4	1.1	12.0
52	Atypical	F	35	L frontotemporal	3.8	1.0	nd
53	Atypical	F	56	R frontal	4.6	1.0	nd
54	Atypical	F	56	L frontal	2.1	0.8	nd
55	Atypical	F	82	R frontal	1.1	1.0	nd
56	Atypical	M	68	L frontal	2.6	0.8	nd
57	Atypical	F	66	L occipital	3.2	0.9	nd
58	Atypical	F	67	R frontal	3.2	1.1	nd
59	Atypical	M	35	L frontal	2.8	0.8	nd
	**Group Mean**		**61**		**3.0**	**1.1**	**12.4**
60	Meningotheliomatous	F	33	R parietal	2.4	1.2	8.2
61	Meningotheliomatous	F	66	Bi parietal	1.0	1.3	15.0
62	Meningotheliomatous	F	66	Bifrontal	1.0	1.4	8.5
63	Meningotheliomatous	M	60	L parietal	3.0	1.4	7.8
64	Meningotheliomatous	F	71	R parietal	1.3	1.0	12.3
65	Meningotheliomatous	F	56	L frontal	1.1	2.0	14.0
66	Meningotheliomatous	F	74	L temporal	1.4	1.8	nd
67	Meningotheliomatous	F	54	L frontal	1.0	1.6	9.9
68	Fibrous	M	87	Bifrontal	1.2	1.9	11.0
69	Fibrous	M	49	L frontal	1.0	4.3	7.2
70	Fibrous	M	78	R frontal	1.0	1.5	15.4
	**Group Mean**		**63**		**1.4**	**1.8**	**10.9**

Supplemental Table 1. Seventy cases divided within three groups based on histology. Demographic information and site of tumor provided. Values for edema index, ADC value and CBV perfusion provided for each tumor. Nd indicates no data available for cases in which a perfusion sequence was not performed. Group means are provided after each group.
